# Usefulness of the heart-rate variability complex for predicting cardiac mortality after acute myocardial infarction

**DOI:** 10.1186/1471-2261-14-59

**Published:** 2014-05-01

**Authors:** Tao Song, Xiu Fen Qu, Ying Tao Zhang, Wei Cao, Bai He Han, Yang Li, Jing Yan Piao, Lei Lei Yin, Heng Da Cheng

**Affiliations:** 1 Department of Cardiology, the First Affiliated Hospital of Harbin Medical University, No.23 Youzheng Street, Nangang District, Harbin City 150001, Heilongjiang Province, China; 2 School of Computer Science and Technology, Harbin Institute of Technology, Harbin, China; 3 Department of Computer Science, Utah State University, Salt Lake City, UT, USA

**Keywords:** Acute myocardial infarction, Cardiac death, Support vector machine, Heart-rate variability, Machine learning

## Abstract

**Background:**

Previous studies indicate that decreased heart-rate variability (HRV) is related to the risk of death in patients after acute myocardial infarction (AMI). However, the conventional indices of HRV have poor predictive value for mortality. Our aim was to develop novel predictive models based on support vector machine (SVM) to study the integrated features of HRV for improving risk stratification after AMI.

**Methods:**

A series of heart-rate dynamic parameters from 208 patients were analyzed after a mean follow-up time of 28 months. Patient electrocardiographic data were classified as either survivals or cardiac deaths. SVM models were established based on different combinations of heart-rate dynamic variables and compared to left ventricular ejection fraction (LVEF), standard deviation of normal-to-normal intervals (SDNN) and deceleration capacity (DC) of heart rate. We tested the accuracy of predictors by assessing the area under the receiver-operator characteristics curve (AUC).

**Results:**

We evaluated a SVM algorithm that integrated various electrocardiographic features based on three models: (A) HRV complex; (B) 6 dimension vector; and (C) 8 dimension vector. Mean AUC of HRV complex was 0.8902, 0.8880 for 6 dimension vector and 0.8579 for 8 dimension vector, compared with 0.7424 for LVEF, 0.7932 for SDNN and 0.7399 for DC.

**Conclusions:**

HRV complex yielded the largest AUC and is the best classifier for predicting cardiac death after AMI.

## Background

Risk stratification of survival after acute myocardial infarction (AMI) could be very useful in guiding post-AMI care, such as implantation of a cardioverter-defibrillator or other optimal medical therapy [1-3]. The degree of left-ventricular ejection fraction (LVEF) impairment is generally used to predict the risk for AMI; however, because most patients exhibit preserved left-ventricular contractile function when acute revascularization procedures are performed after AMI, the ability to stratify risk based on LVEF is reduced. Several previous studies have indicated that decreased heart-rate variability (HRV), a marker of cardiac autonomic nervous system dysfunction, is associated with a high risk for severe ventricular arrhythmias and mortality in the post-AMI population [4]. However, conventional methods to analyze HRV, such as standard time and frequency domain measures, have relatively poor accuracy for predicting outcomes with current treatment strategies [5-7]. Recent Holter-based predictive variables, such as heart-rate turbulence and deceleration capacity (DC) of heart rate, have demonstrated greater prognostic power in post-AMI patients; however, the clinical usefulness is not well established. HRV methods can capture several aspects of autonomic heart rate modulation, but individual HRV indices do not reflect global effects of autonomic nervous system dysfunction on the disease. This may be the cause of the limited predictive capacity of HRV approaches. Therefore, a comprehensive method to quantify the global effects of the autonomic nervous system on heart rate modulation and to provide individualized risk stratification in the post-AMI population is desperately needed. In this study, we developed a support vector machine (SVM) algorithm based on heart-rate dynamics that measures the relationship between autonomic nervous dysfunction and AMI mortality. We used this novel analytical tool to more accurately classify high-risk AMI patients. We postulate that the integrated features of HRV are better than individual measures of HRV and DC at predicting mortality risk in the post-AMI human population.

## Methods

### Participants

For this study, 226 patients aged from 18 to 80 years who were admitted for AMI to the Cardiology Department of the 1st hospital of the Harbin Medical University in Harbin, China from January 2009 to December 2009 were enrolled. AMI was diagnosed at the time of admission by at least 2 of the following findings: chest pain for ≥20 min; creatine kinase-MB more than double the upper normal limit of our laboratory; ST-segment elevation ≥0.1 mv in at least two limb leads or ≥0.2 mv in at least two contiguous precordial leads; and primary motion abnormality of the left ventricular regional wall in the echocardiography or MRI examination. We excluded patients if they: (1) had severe valvular, pulmonary, hepatic, renal or other severe concomitant noncardiovascular disease; (2) were logistically unable to participate; (3) exhibited sinus rhythm for <80% of the total 24-hour Holter recording; (4) had atrial flutter, atrial fibrillation or pacemaker rhythm; (5) had inadequately analyzable electrocardiographic data; and/or (6) had inadequate follow-up data. Furthermore, all of patients underwent routine cardiac medication and AMI treatment according to the contemporary guidelines [8].

The analysis of electrocardiographic data and the monitoring of patients were approved by the Institutional Ethics Committee for human research of the Harbin Medical University. Since the data collection was noninvasive and did not affect routine clinical management of the patients, the Ethics Committee deemed oral informed consent to be sufficient for patient participation.

### Procedures

Holter electrocardiograms were recorded for 24 hours during the second week after infarction (average 8–14 days) for patients that were stable during this period. The recordings were digitized at 128 Hz, automatically processed with a GE Holter system (GE medical, USA), and scanned with GE Holter analysis software following standard procedures as specified by the manufacturer. Manual verification of uncorrected QRS classifications and reduction of noise was conducted as needed. Meanwhile, we assessed LVEF by single-plane echocardiography one week after index infarction, with <0.35 predefined as seriously abnormal. Other risk predictors included age, history of myocardial infarction, diabetes mellitus, hypertension, and medical treatments, which were determined by the physician using a standardized protocol.

### Automatic feature extraction

Machine learning algorithms require data to be represented by features, such as the indices that occur in an electrocardiographic recording. In this study, a total of 10 indices were extracted from every Holter recording. The electrocardiographic features of the patients are shown in Table 1 and include the following: indices of HRV; heart-rate turbulence; mean heart beat; DC; and acceleration capacity of heart rate. As proposed by the Task Force of the European Society of Cardiology and the North American Society of Pacing and Electrophysiology [9], we calculated the following conventional risk predictors from time domain measures of HRV in the 24-hour Holter recordings. Time domain measures of the R-R time series were used to quantify the variability of the R-R intervals, including standard deviation of all normal-to-normal intervals [SDNN (ms)], average standard deviation of all normal-to-normal intervals (ms), standard deviation of average normal-to-normal intervals (ms), mean normal-to-normal intervals (ms), and triangle index of geometric figure parameter of the R-R time series. The spectral measures of HRV were analyzed using the methods recommended by the Task Force, and the cut-off values of reduced spectral indexes were also predefined. These traditional indices of HRV have a proven ability to predict mortality [10] and are easy to calculate. High resting heart rate has been associated with cardiac death [11]. In this study, mean heart rate was calculated as heart beat number per hour. We assessed other newer risk predictors of heart-rate dynamics as well. Heart-rate turbulence indices, including turbulence onset and turbulence slope (ms), reflect the baroreflex mediated short-term oscillation of cardiac cycle lengths after spontaneous ventricular premature complexes. These were calculated in the instances when ventricular premature beats were found in the Holter recordings [12]. Turbulence slope was defined as the maximum slope of the regression line assessed over any sequence within five subsequent sinus R-R intervals during the first fifteen sinus heart beats following an ectopic ventricular beat. A decreased heart rate turbulence is related to autonomic dysfunction and has a powerful ability to predict sudden cardiac death [13]. Two newer Holter-based risk variables, which are developed by a phase rectified signal averaging technique—namely, DC (ms) and acceleration capacity (ms) of heart rate—were calculated to provide a separate assessment of autonomic modulation of the heart rate. DC reflects the regulation of the vagus nerve on heart rate. This is of clinical importance because vagus withdraw has been regarded as an important mechanism of sudden death [14]. Furthermore, these features are all thought to reflect functional changes of the autonomic nervous system.

**Table 1:** Electrocardiographic features of heart-rate dynamics extracted from Holter recordings

**Methods**	**Features**
Time domain of HRV	Standard deviation of all normal-to-normal intervals
	Average standard deviation of all normal-to-normal intervals
	Standard deviation of average normal-to-normal intervals
	Mean normal-to-normal intervals
	Triangle index of a geometric figure
Counting of R-R series	Mean heart rates per hour
Heart rate turbulence	Turbulence onset
	Turbulence slope
Phase rectified signal averaging	Heart rate deceleration capacity
	Heart rate acceleration capacity

### SVM algorithm

In machine learning, SVM are supervised learning models with associated learning algorithms that analyze data and recognize patterns. SVMs are used for classification and regression analyses, and the classification strongly depends on the available feature set and the tuning of hyper-parameters [15]. We used SVMs to analyze the predictive power of the combined indices from every Holter recording. In this study, SVM was developed to maximize the margin of the hyper-plane dividing electrocardiographic data into two classes, survivals and cardiac deaths, by finding the best hyper-plane that separates clusters of features represented in an n-dimensional space. A hyper-plane can be written as the set of points X satisfying W^T^X + b = 0, where W^T^ is a normal vector perpendicular to the hyper-plane, X is the vector of electrocardiographic features, and b is bias or offset of the hyper-plane from the origin. Inputs of SVM are mapped onto a high dimensional feature space via kernel functions, and the optimal hyper-planes are constructed to separate samples into two classes [16]. SVMs were trained and tested using the leave-one-out and cross-validation methods, which were applied to evaluate the accuracy of classification. At every step, one electrocardiographic data subset was left out from the total training dataset and treated as undefined data. The classification model was constructed with the remaining data and the algorithm classified the “left-out” electrocardiographic data. The same procedure was applied to the training dataset. The results of SVM were normalized in the range from 0 to 1. We prospectively defined a cut-off point as >0.5 indicated higher probabilities of death, while <0.5 indicated higher probability of survival. Through training and testing, we finished feature selection and searched for the optimal vector that had the greatest predictive power.

At the same time, comparisons were made with LVEF, SDNN and DC using the identical dataset. The SDNN measured from the 24-hour R-R intervals was chosen as a conventional index of HRV, with a value <70 ms predefined as abnormal [10]. Meanwhile, DC measured from the 24-hour recording was chosen as a novel index of heart-rate dynamics, with a value <4.5 ms predefined as abnormal [14].

### Follow-up and endpoints

Patients with AMI underwent physical examination 6 months after discharge and annually thereafter by telephone assessment. The patients were followed for a mean of 28 months after AMI. In cases of death, the causes were verified from the hospital records, death certificates, autopsy records and telephone interviews with either the primary physicians or those who had witnessed the death. An independent endpoint committee determined the cause of death according to the available data from the aforementioned sources. Witnessed deaths due to cardiac diseases including myocardial infarction, heart failure, severe malignant arrhythmia and those occurring after attempted resuscitation by defibrillator were all classified as cardiac death. Unwitnessed deaths occurring in a previously asymptomatic patient with no other life-threatening diseases was classified as cardiac death as well. Based on these determinations, the electrocardiographic data were then classified as survivals or cardiac deaths. To avoid bias, the computer center received only electrocardiographic data and classification results without other clinical information of patients.

### Statistical analysis

We calculated receiver-operator characteristic curves to evaluate prediction accuracy of SVM models, LVEF, and conventional risk indices of HRV and DC. We quantified receiver-operator characteristic curves by taking the integrals of the curves (area under the curve; AUC), with the continuous variable being set to a plurality of different critical values, to calculate a series of sensitivity and specificity thresholds. The sensitivity was used as the ordinate and negative positive rate (1- specific) as the abscissa to plot the curves. The AUC of a method was prospectively defined as the statistical measure of the predictive power. The accuracy, sensitivity, specificity, positive predictive value and negative predictive value of each variable were taken into account [17]. The difference between two receiver-operator characteristic curves was compared by non-parametric test, with a P value less than 0.05 considered statistically significant.

## Results

Based on initial evaluation with the GE analysis software, 6 electrocardiograms were excluded because of non-cardiac death and 12 electrocardiograms were excluded because of unacceptable deviations in the recordings or otherwise inadequately analyzable data. Thus, a total of 208 patient dynamic electrocardiograms were included in the subsequent analyses. Among these data, 196 (94.2%) recordings were classified as survivals and 12 (5.8%) recordings were classified as cardiac deaths, including 7 sudden cardiac deaths and 5non-sudden cardiac deaths. Among these patients, 207 of the 208 patients were used as the “training” subjects and the remaining patient was used as a “test” subject. Repeating the dataset from the test subject 208 times, the classification accuracy was then calculated by the number of correctly classified patients divided by the total number of patients. The clinical characteristics of the patients are shown in Table 2. For the treatment, 112 patients (53.8%) underwent PTCA, and thrombolysis was performed in 45 patients (21.6%). In addition, other treatments such as β-blockers, ACE inhibition or ARBs were used in more than 75% patients, and aspirin or statins were used in more than 97% patients. Therefore, most patients in this study received modern standard of care for AMI.

**Table 2:** Patient baseline characteristics and treatment

**Clinical features**	**Survivals (n = 196)**	**Cardiac deaths (n = 12)**
**Study characteristics**		
Follow-up (months)	27.99 ± 6.02	11.17 ± 10.85
**Patient characteristics**		
Age (years)	60.19 ± 11.39	71.83 ± 7.29*
Sex (female)	52 (27%)	6 (50%)*
LVEF	54.86 ± 11.18	46.00 ± 11.15*
LVEF < 0.35	9 (4.6%)	3 (25%)*
Smoking (ex or current)	110 (56%)	4 (33%)*
Diabetes mellitus	42 (21%)	8 (67%)*
Previous myocardial infarction	25 (13%)	2 (17%)
Hypertension	95 (48%)	11 (92%)*
**Treatment**		
PTCA	117 (60%)	7 (58%)
Thrombolysis	44 (22%)	2 (17%)
No acute revascularization	35 (18%)	3 (25%)
Aspirin or clopidogrel	194 (99%)	12 (100%)
β-blockers	158 (81%)	10 (83%)
Statins	192 (98%)	10 (83%)*
ACEI/ARB	150 (77%)	9 (75%)
Diuretic	134 (68%)	7 (58%)

Data are number (%) unless otherwise stated. *P < 0.05.

Among the established 10 electrocardiographic features we evaluated, heart-rate turbulence indices depended on the existence of ventricular premature beats. However, a ventricular premature beat might not have existed in every recording, a total of 83 recordings have no ventricular premature in this cohort. Inherent to the procedures of machine learning, a missing feature in the dataset was difficult to accommodate in the analyses. To avoid biases according to this consideration, the features with possible missing data points were not employed. As a result, heart-rate turbulence could not be used as a classifier in this study. Ultimately, 8 features of extracted heart-rate dynamics indices were used to construct SVM models. The electrocardiographic features of the patients after feature selection and the differences between the two classes are shown in Table 3.

**Table 3:** Electrocardiographic characteristics of the patients, after feature selection, that were used to construct SVM predictive models

**Variables**	**Survivals (n = 196)**	**Cardiac deaths (n = 12)**	**P value**
Standard deviation of all normal-to-normal intervals	96.22 ± 27.86	64.52 ± 14.65	<0.001
Standard deviation of average normal-to-normal intervals	83.66 ± 26.65	58.47 ± 15.68	0.001
Average standard deviation of all normal-to-normal intervals	43.40 ± 13.67	25.74 ± 6.84	<0.001
Mean normal-to-normal intervals	893.54 ± 115.46	826.85 ± 84.21	0.051
Triangle index (number%)	27.48 ± 8.81	16.80 ± 4.20	<0.001
Heart rate deceleration capacity	6.31 ± 1.91	4.21 ± 1.44	<0.001
Heart rate acceleration capacity	-6.27 ± 1.98	-4.12 ± 1.42	<0.001
Mean heart rates per hour (number)	68.36 ± 9.00	73.50 ± 7.70	0.054

Data are ms unless otherwise specified.

Linear predictive models are in most cases ineffective in solving death prediction problems. To circumvent this challenge, we searched for the kernel’s parameters that could be optimized to obtain the best classification accuracy. This optimized first employed training and testing the technique to check each combination of parameter choices. Then, the parameters that resulted in the best accuracy were selected. Next, the final models were trained on the whole training set using the chosen parameters. The result was a 5 dimension HRV feature vector, which is called a HRV complex. In this study, we constructed 3 SVMs using different combinations of the features that performed better than other combinations tested: (A) HRV complex (5 time domain measures of HRV); (B) 6 dimension vector (HRV + DC + acceleration capacity, with mean normal-to-normal intervals removed); and (C) 8 dimension vector (HRV + DC + acceleration capacity + mean heart rate). The results of accuracy, sensitivity, specificity, positive predictive value and negative predictive value of SVMs are shown in Table 4. Although SVMs had an accuracy rate of only 0.80, they had greater sensitivities and better negative predictive values. Among the SVMs, the sensitivities of HRV complex and 6 dimension vector both reached 0.91, whereas SDNN had sensitivity of only 0. 58. The results of sensitivity, specificity, positive predictive value and negative predictive value of the HRV complex, SDNN and DC in subgroup with LVEF > 0.35 are shown in Table 5. The HRV complex had better sensitivity, positive predictive value and negative predictive value as compared with SDNN and DC in these patients.

**Table 4:** Accuracy, sensitivity, specificity, positive predictive value and negative predictive value of HRV complex, 6 dimension, 8 dimension, LVEF, SDNN and DC (%)

**Methods**		**Accuracy**	**Sensitivity**	**Specificity**	**Positive predictive value**	**Negative predictive value**
	HRV complex	79.81	91.67	79.08	21.15	99.36
SVM	6 dimension	79.33	91.67	78.57	20.75	99.35
	8 dimension	79.33	83.33	79.08	19.61	98.73
			LVEF	91.83	25.00	95.41	25.00	95.41
			SDNN	83.65	58.33	85.20	19.44	97.09
			DC	80.77	50.00	82.65	15.00	96.43

**Table 5:** Sensitivity, specificity, positive predictive value and negative predictive value of HRV complex, SDNN and DC in patients with LVEF > 0.35 (%)

**Methods**	**Sensitivity**	**Specificity**	**Positive predictive value**	**Negative predictive value**
**HRV complex**	88.89	80.21	17.78	99.34
**SDNN**	55.56	86.63	16.67	97.59
**DC**	55.56	82.89	13.51	97.48

**Figure 1:**
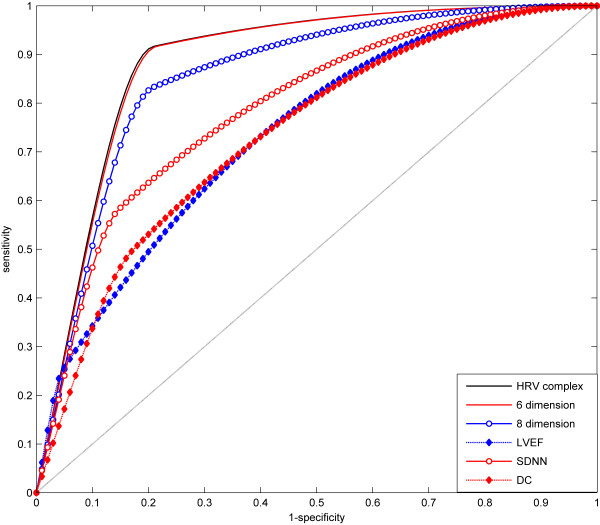
**Receiver-operator characteristic curves for predicting cardiac deaths by HRV complex, 6 dimension, 8 dimension, LVEF, SDNN and DC.** Sensitivity is determined from the proportion of cardiac deaths identified as high risk; specificity is determined from the proportion of survivors identified as low risk.

Receiver-operator characteristic curves for predictions of SVM models are shown in Figure 1. The mean AUCs of receiver-operator characteristic curves are shown in Table 6. In SVM models, AUC of the HRV complex (0.8902) was larger than that of the 6 dimension vector (0.8880) and the 8 dimension vector (0.8579). Compared to traditional indices, the AUCs of the SVM models were significantly larger than those of LVEF (0.7424, p < 0.01), SDNN (0.7932, p < 0.05) and DC (0.7399, p < 0.01). Hence, SVM predictive models based on heart-rate dynamics variables yield larger AUCs than LVEF, SDNN and DC.

**Table 6:** Average AUCs of ROC of HRV complex, 6 dimension, 8 dimension, LVEF, SDNN and DC

**Methods**	**AUC**	**P value**
**SVM**		
** HRV complex**	0.8902	-
** 6 dimension**	0.8880	>0.05
** 8 dimension**	0.8579	>0.05
** LVEF**	0.7424	<0.01
** SDNN**	0.7932	<0.05
** DC**	0.7399	<0.01

## Discussion

In this study, we used noninvasive heart-rate dynamics indices extracted from electrocardiographic datasets from patients following AMI to develop a computer-aided predictive tool that improves risk stratification. By combining the predictive power of multiple heart-rate dynamics variables, SVM models are an effective approach to quantify the risk of cardiac death and are capable of prospectively identifying high-risk patients in the post-AMI population. The HRV complex we describe in this study provides a novel analysis method to evaluate the effect of autonomic nervous system dysfunction on cardiac death following AMI. Moreover, this new predictive strategy has an enhanced discriminating threshold for AMI as compared to current electrocardiographic and echocardiographic methods (i.e. LVEF, SDNN and DC).

Autonomic nervous system dysfunction has a significant adverse effect on outcomes in post-AMI patients. Therefore, quantitative assessment of autonomic dysfunction could enhance the predictability of cardiac death [18,19]. The effects of autonomic modulators on the heart can be quantified by noninvasive HRV methods, and conventional indices of HRV developed by simple calculation of R-R series have demonstrated prognostic value for mortality [20]. Nevertheless, individual HRV indices do not reflect the combined effects of the autonomic nervous system on heart rate, and therefore these traditional measures have only a partial predictive capability. In this study, to advance our ability to predict outcomes in AMI patients, individual HRV indices were regarded as the features of high-risk of cardiac death, and a SVM was used to combine the predictive power of multiple HRV indices. As a result, we developed a novel HRV analysis method, termed HRV complex, which was effective at predicting cardiac death after AMI. Whether our method will be effective to quantify entire autonomic nervous system dysfunction will require additional studies. Nevertheless, our results clearly show that the HRV complex has a better prognostic capacity compared with SDNN or DC of heart rate. The HRV complex also outperforms the combination of HRV indices and DC. In this sense, the HRV complex is a better prognostic tool than individual HRV indices, and the classifiers that incorporate the combined effects of vagal and sympathetic modulators are better than a single nerve modulator.

An ideal risk stratification tool needs to be accurate, cost effective and have the capacity to reduce the risk of death across different patient populations [21,22]. In this study, the specificity of LVEF is higher than other methods; however, the sensitivity is quite low. Due to the fact that the number of survivals and cardiac deaths are unbalanced, the higher specificity does not reflect the capacity of classification. This leads to a lower predictive value than a tool based on LVEF. To overcome this limitation, machine-learning approaches, which utilize “training” and “testing” exercises with a well-characterized dataset, were used. SVM is a good learning method that is well suited for small samples, and it does not involve probability measures. Thus, SVM is different from the existing statistical methods and not easily susceptible to over-fitting. SVM uses insights from training samples to forecast probabilities and greatly simplifies the classification and regression problems. In this study, we used the leave-one-out and cross-validation method to carry out “training” and “testing.” this method achieves most of the benefits of machine learning [23]. In the subgroup of patients with LVEF > 0.35, the sensitivity of HRV complex is about 0.89, meaning that the HRV complex could recognize ~89% of high risk patients which were otherwise identified as low risk by LVEF.

To verify the HRV complex’s usefulness for the discrimination of patients likely to benefit from post-AMI therapy, the SVM model was designed to test the ability of HRV variables to stratify an AMI patient population. In the present study, we used cardiac death, which may arise clinically as the result of an autonomic disorder after severe cardiovascular damage (e.g. a fall in blood pressure eliciting a surge in sympathetic outflow), as the main endpoint. The HRV complex has several potential advantages over a single stratification tool. First, it combines predictive variables and is therefore more accurate. Through “training” and “testing”, the SVM predictive model combines the prognostic power of several established stratification tools. Second, the HRV complex provides a digital risk estimate rather than a binary distinction of just “high” or “low” risk. This quantified risk assessment can aid clinicians in determining optimal cost-effective preventive strategies. Finally, the HRV complex reflects the underlying mechanism of cardiac death after AMI, and as a result will better predict outcomes as compared with current methods. We suspect that autonomic dysfunction as the trigger of cardiac death could be determined by our method. Thus, this stratification tool could improve the discrimination of patients eligible for cardioverter-defibrillator implantation [24].

We assessed the accuracy of prediction by our model by calculating the AUCs of the receiver-operator characteristic curves. This method accounts for dependency of specificity on sensitivity and is independent of specific cut-off values. Classifiers with AUCs > 0.80 are considered good in classification [25]. AUCs of methods A, B, and C in our study of SVMs were all higher than 0.80, demonstrating their strong predictive abilities. The HRV complex yielded the largest AUC (0.8902), exhibiting the largest prognostic capability, which was slightly larger than the 6 or 8 dimension vector models (0.8880 and 0.8579, respectively). This is likely because some features, such as mean heart rate, mean N-N intervals or acceleration capacity of heart rate, have relatively small effects on the distinction between high and low-risk populations. In the present population studied, the largest separation of receiver-operator characteristic curves between HRV complex and SDNN or DC had a sensitivity level of about 90%. The HRV complex-based risk assessment was especially suitable for accurate identification of high-risk patients who would benefit from further interventions or device implantation. Impaired DC of heart rate has been reported as a powerful predictor of all-cause mortality after AMI [14]. However, it did not play an important role in this study. This may be due to the sample size in the present study and/or because DC is not suitable to specifically predict cardiac (as opposed to all-cause) death after AMI. The accuracy of SVMs was quite good, but the size of the training dataset was small and the number of deaths was small. Hence, it is necessary to evaluate how a larger training set affects the predictive accuracy of SVMs in future studies.

There are several limitations to this study. First, we only use traditional established risk predictors of HRV to construct predictive models without including other strong risk markers, such as some nonlinear measurements of heart rate dynamics and T wave alterations [26,27]. The addition of these markers may provide incremental predictive power. Second, we employed HRV indices from 24-hour Holter recordings and did not determine the difference of risk classification by the HRV complex during daytime versus nighttime. We only obtained a single Holter recording in the second week after AMI; it is possible that serial or more frequent measurements would reveal differential risk amongst these patients. Third, whether the proposed SVM models can perform well using other datasets remains unknown. Therefore, this novel predictive algorithm should be studied in a larger number of patients. Fourth, we used cardiac death as the main endpoint. Although sudden cardiac death includes a subset of patients who succumb to lethal arrhythmias and who would therefore benefit from prophylactic interventions, no universally accepted definition for sudden cardiac death is available. Thus, we could not estimate how many of the patients in our study would be candidates for device therapy to prevent/respond to arrhythmias. In this study, we showed that the HRV complex was a useful risk predictor following AMI. However, we have no data to show whether specific treatments in response to such a predictor will improve patient outcomes. This goal will be pursued in a future study.

## Conclusions

This study identifies a novel risk predictor that we have termed the HRV complex, which improves stratification of post-AMI patients. The predictive accuracy of HRV complex is better than LVEF, individual established indices of HRV and DC of heart rate. The performance suggests that the HRV complex is an accurate, non-invasive, quantitative screening metric for improving risk stratification of the post-AMI population. It has great potential to provide a framework to aid clinical decision-making in the prediction of cardiac deaths.

## Competing interests

The authors declare that they have no competing interests.

## Authors’ contributions

TS, XQ, YL and HC have substantial contributions to conception and design. TS, YZ, WC, BH, JP and LY have participated in acquisition of data, or analysis and interpretation of data. TS, XQ, YZ, YL and HC have been involved in drafting the manuscript and revising it. All authors read and approved the final manuscript.

## Pre-publication history

The pre-publication history for this paper can be accessed here:

http://www.biomedcentral.com/1471-2261/14/59/prepub
